# Association between extracellular signal-regulated kinase expression and the anti-allodynic effect in rats with spared nerve injury by applying immediate pulsed radiofrequency

**DOI:** 10.1186/s12871-015-0071-3

**Published:** 2015-06-16

**Authors:** Chun-Chang Yeh, Zhi-Fu Wu, Jui-Chieh Chen, Chih-Shung Wong, Chi-Jung Huang, Jinn-Shyan Wang, Chih-Cheng Chien

**Affiliations:** 1 School of Medicine, Fu Jen Catholic University, New Taipei, 24205 Taiwan; 2 Department of Anesthesiology & Integrated Pain Management Center, Tri-Service General Hospital and National Defense Medical Center, Taipei, Taiwan; 3 Department of Chemistry, Fu-Jen Catholic University and Graduate Institute of Basic Medicine, Fu-Jen Catholic University, Taipei, Taiwan; 4 Department of Biochemical Science and Technology, National Chiayi University, Chiayi, Taiwan; 5 Department of Medical Research, Cathay General Hospital, Taipei, Taiwan; 6 Department of Anesthesiology, Sijhih Cathay General Hospital, New Taipei, Taiwan; 7 Department of Anesthesiology, Cathay General Hospital, Taipei, Taiwan; 8 Department of Biochemistry, National Defense Medical Center, Taipei, Taiwan

**Keywords:** Pulsed radiofrequency, Spared nerve injury, ERK1/2, Neuropathic pain

## Abstract

**Background:**

The application of pulsed radiofrequency (PRF) close to the dorsal root ganglia, or peripheral nerves, has been demonstrated to be effective for the treatment of chronic neuropathic pain conditions. The goal of this study was to investigate the analgesic effect of immediate PRF treatment after nerve injury and its possible cellular alterations in the dorsal horn of the spinal cord in rats with spared nerve injury (SNI).

**Methods:**

Neuropathic pain was achieved in a SNI neuropathic pain model by ligating and cutting the common peroneal and tibial branches of the left sciatic nerve, leaving the sural nerve intact. Wistar rats were divided into four groups that received different treatments, i.e., SNI and PRF for 6 min at 45 V (SNI + PRF-45 V), at 60 V (SNI + PRF-60 V), SNI alone, and sham groups. After the SNI surgery, each rat was immediately given the PRF treatment (500 kHz, rate of 2 Hz, 20 ms duration, temperature below 42 °C) on the left sciatic nerve 0.3–0.4 cm proximal to the injured site. The behavioral measurements included mechanical allodynia and cold allodynia of the ipsilateral hind paw and were performed during the 28 days that followed the SNI surgery and PRF treatment. Total extracellular signal-regulated kinase 1 and 2 (ERK1/2) and phospho-ERK1/2 were measured using Western blot in the ipsilateral spinal cord from animals in the different groups.

**Results:**

The three groups of rats with nerve injuries manifested a lower paw withdrawal threshold (PWT) in the behavioral measurement of mechanical allodynia and a shorter painful-behavior duration in the cold allodynia test over 28 days. Mechanical allodynia measurement showed that both the PRF-45 V and PRF-60 V treatment groups exhibited a more prominent antiallodynic effect than did the SNI group from days 1 to 28 after surgery. Similarly, in comparison with the SNI group, both the SNI + PRF-45 V and SNI + PRF-60 V groups had significant inhibition on the cold allodynia measurement from days 1 to 28 after surgery. Furthermore, the activation of the extracellular signal-regulated kinase 1 and 2 (ERK1/2) in the ipsilateral spinal dorsal horn of SNI rats was effectively inhibited in the SNI + PRF-45 V and SNI + PRF-60 V groups for 28 days after surgery.

**Conclusions:**

Immediate PRF application on the proximal nerve injury site provided a significant inhibition of neuropathic pain formation, accompanied by the inhibition of ERK activation.

## Background

Neuropathic pain, which is a result of central or peripheral nerve damage, is characterized by a complex constellation of unusual pain symptoms, such as spontaneous and evoked pain (allodynia and hyperalgesia) [1–3]. The clinical application of all forms of pharmacotherapy provides only partial reduction of pain, and frequently there is unsatisfactory pain relief with many unwanted side effects [4]. Pulsed radiofrequency (PRF) treatment was introduced in 1996 [5] and has been used as an effective therapeutic technique for chronic refractory pains, including postamputation stump pain, cervical and lumbar radicular pain, shoulder and knee arthropathic pain, and some neuropathic pains. It has been shown to be safe and helpful, without clinical evidence of nerve damage compared with conventional radiofrequency thermocoagulation [6–14].

PRF therapy delivers a brief high-frequency electrical stimulation adjacent to the dorsal root ganglia (DRG) or a sensory nerve without causing further tissue injury [15]. Aksu *et al.* first demonstrated that both thermal and mechanical hyperalgesia were attenuated by PRF application on L5 and L6 dorsal roots in rabbits with sciatic nerve injury (neuropathic pain model) [16]. Perret *et al*. also observed that PRF provided a significant reduction of allodynia and mechanical hyperalgesia for more than 32 days post-PRF treatment delivered adjacent to the DRG in rats with spinal nerve injury [17].

The mechanism underlying the analgesic effect of PRF treatment remains unclear. PRF application at DRG may alter the biological actions on synaptic transmission, cell morphology, and Fos expression in the superficial dorsal horn of the spinal cord, with trivial effects on nerve tissues [18–20]. Furthermore, PRF may augment noradrenergic and serotonergic descending inhibitory systems within the spinal cord, which afforded an analgesic effect in an inflammatory pain model [21]. Evidence has shown that extracellular signal-regulated kinase (ERK) plays a decisive role in regulating inflammatory responses and neuropathic pain [22, 23]. A recent study also reported that ERK knockout mice exhibited decreased pain sensitization after formalin stimulation or partial sciatic nerve ligation [24]. Thus, inhibition of ERK activation may be a promising therapeutic target for the treatment of neuropathic pain [25–27]. In the present study, we examined the analgesic effect of PRF treatment at the usual clinical conditions (PRF waves with a 500 kHz frequency, 45 or 60 V output, and 20 ms pulse width; delivered for 6 min) and the relationship between PRF and ERK1/2 expression in the dorsal horn of the spinal cord in rats with spared nerve injury (SNI).

## Methods

### Animals, Setting, and Ethics

This study was approved by the Animal Care and Use Committee of the National Defense Medical Center (Taipei, Taiwan, Republic of China) and was conducted in accordance with the Guide for the Care and Use of Laboratory Animals published by the National Institutes of Health (Bethesda, Maryland).

Male Wistar rats (BioLASCO, Taipei, Taiwan) weighing 200–250 g were housed individually with soft bedding in a 12 h night/day cycle with free access to food and water at all times in a similar environment for 7 days, for acclimation before SNI surgery. All efforts were made to minimize the number of animals used and their suffering. Rats were randomly divided into four groups that were treated with or without PRF for 6 min (3 min per session, with a 30 s intersession interval): SNI + PRF-45 V group, SNI + PRF-60 V group, SNI group (RF needle was put on but no current was delivered), and sham-operated + placebo PRF group. Animals were assessed for mechanical allodynia using dynamic plantar aesthesiometry (DPA) and for cold allodynia using the acetone spray test 1 day before surgery (baseline), without clinical evidence of nerve damage or various days (at day 1 (D1), D3, D7, D10, D14, D21, and D28) after surgery, as indicated in the single PRF treatment.

### Neuropathic pain Model

The neuropathic pain model was induced by SNI described by Decosterd and Woolf [28]. SNI was performed under 1.5 % isoflurane (Halocarbon, NJ, USA) anesthesia. A 2–4 mm ligated nerve of the common peroneal and tibial branches of the left sciatic nerve was removed, and sural nerve was left intact. Sham surgery refers to the same protocol but without nerve injury.

### Assessment of Antinociception to Pulsed Radiofrequency

**Fig 1:**
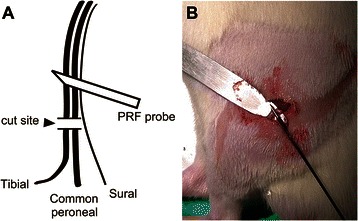
Application of pulsed radiofrequency (PRF) in a model of spared nerve injury (SNI). **a**. Diagram. **b**. Actual application of PRF immediately after SNI surgery

All rats were randomly divided into four groups (*n* = 7 for each group). PRF was administered via an electrocautery disk placed in a right decubitus position and connected to the PRF generator (NeuroTherm, NT1000, UK). The 5 mm active tip electrode (NeuroTherm 22 GA) was placed vertically, adjacent to the left sciatic nerve (0.3–0.4 cm proximal to the injury site) immediately after the SNI surgery (Fig. 1). PRF treatment (500 kHz) with an output of 45 or 60 V or no output was delivered at a rate of 2 Hz, 2 bursts/s with a 20 ms duration for 6 min at a temperature below 42 °C. The SNI group and sham-operated + placebo group were used as controls, respectively. The former was achieved by placing the electrode 0.3–0.4 cm proximal to the injury site, whereas the latter was performed by placing the electrode on an intact sciatic nerve. The procedures performed in both groups were identical to those used in the PRF treatment groups, but without application of an electric current. The probe was lightly placed in a perpendicular direction to the sciatic nerve, and the temperature of the tip of the electrode was controlled below 42 °C. At the end of the procedure, the skin incision was closed with 4-O silk sutures, and the animal was allowed to recover from anesthesia.

### Behavioral Testing of Tactile Allodynia

Mechanical allodynia was examined using a dynamic plantar aesthesiometer (DPA; Ugo Basile, Comerio, Italy), which is an automated version of the von Frey filament that does not produce tissue damage [29, 30]. According to the Kyoto protocol of the International Association for the Study of Pain, Basic Pain Terminology, the DPA produces non-noxious tactile stimuli [31]. Each rat was placed in an individual plastic cage (25 cm long × 10 cm wide × 14 cm high) with a wire mesh floor, was acclimatized to the cage for 15 min before each test session, and a paw withdrawal response was elicited by applying an increasing force using a blunt-end metal filament (0.5 mm in diameter) focused on the territory of the sural nerve at the palmar surface of the left ipsilateral hind paw. The force was increased from 1 to 50 g in steps of 1 *g* over 20 s, and was then held at 50 *g* for an additional 10 s; the rate of the force increase was 2.5 *g*/s. The threshold was recorded as the force that elicited the hind paw removal reflex (the mean of three measurements performed at 1 min intervals).

### Cold Allodynia Behavioral Testing

Cold hypersensitivity was determined by measuring the cold withdrawal response of the hind paw to an acetone spray. Rats were placed in a transparent plastic cage on top of a wire mesh grid, which allowed access to the paws, and were adapted to the testing environment for 15 min before the measurement was performed. Cold allodynia was assessed by spraying acetone (100 μL) using an Eppendorf multistepper pipette onto the palmar surface of the ipsilateral hind paw through the wire mesh floor, and the duration of shaking, flinching, biting, or licking behavior that ensued in a 1 min period was measured [32, 33]. Each rat was tested five times with a minimal interval of 5 min. A minimal value of 0.5 s was given if there was a fast or brisk reaction, whereas a value of 0 was given if no reaction was observed at all.

### Spinal Cord Preparation and Western Blotting Analysis

All rats underwent behavioral tests of mechanical allodynia and cold allodynia and were killed immediately after the tests, at different days of completion. The rats were rapidly decapitated and the left dorsal quadrant of the lumbar spinal cord enlargement was removed and stored at −80 °C until use. Tissue samples from the L4–L6 dorsal horns were lysed by homogenizing in 200 μL of lysis buffer (30 mM Tris, 2 M thiourea, 7 M urea, 4 % CHAPS, protease inhibitor cocktail (Merck 1:100), and phosphatase inhibitor cocktail set V (Merck 1:50) adjusted to pH 7.4 with HCl). Lysates were centrifuged at 20,000× *g* for 20 min at 4 °C and the protein concentration in each sample (supernatant) was determined using the 2-D Quant kit (GE Healthcare). Samples with an equal amount of protein were then separated by SDS–PAGE and transferred to PVDF membranes (Millipore, Bedford, MA). The membranes were blocked with blocking buffer (20 mM Tris–HCl, pH 7.5, 150 mM NaCl, and 5 % skim milk) for 1 h at room temperature with gentle shaking, followed by washing with TNT buffer (20 mM Tris–HCl, pH 7.5, 150 mM NaCl, and 0.05 % Tween 20) and overnight incubation at 4 °C with mouse anti-p-ERK antibodies (Santa Cruz Biotechnology) diluted at 1:200 in TNT buffer. After washing with TNT buffer, the membranes were incubated for 1 h at room temperature with an HRP-conjugated anti-mouse IgG antibody (BD Biosciences Pharmingen). Bands were visualized using enhanced chemiluminescence reagents (Millipore), and images were recorded on a computer using the UVP Bioimaging System (UVP, Upland, CA, USA). After stripping, the membranes were reprobed with rabbit anti-ERK antibodies (Santa Cruz Biotechnology) diluted at 1:200 in TNT buffer. The membranes were then incubated for 1 h at room temperature with an HRP-conjugated anti-rabbit IgG antibody (GeneTex Inc.). The final detection was performed as described above.

### Statistical Analyses

Data are presented as the mean ± standard deviation (SD). We used linear contrasts to calculate *P* values. The first contrast was used to compare the “SNI effect”: we set the assign coefficients as 1, −1/3, −1/3, and −1/3 for the sham, SNI, SNI + PRF-45 V, and SNI + PRF-60 V groups, respectively. The second contrast was used to compare the “treatment effect”: we set the assign coefficients as 0, −1, −1/2, and −1/2, respectively. The third contrast was used to compare the “dosage effect”: we set the assign coefficients as 0, 0, 1, and −1, respectively. Data pertaining to the relative density of western blot bands were analyzed by one-way ANOVA, followed by multiple comparisons using the Student–Newman–Keuls post hoc test. Data from behavioral tests are presented as the mean ± SD. Data pertaining to the quantitative densitometry of the pERK1/2 to ERK1/2 expression ratio were expressed as the mean ± standard error of the mean (SEM). We considered *P* < 0.05 as significant in all analyses. Statistical analyses were carried out using the R3.1.1 software (“multicom” package).

## Results

**Fig 2:**
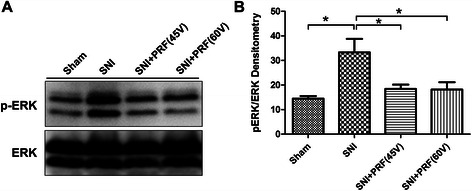
Paw withdrawal test of mechanical allodynia. The paw withdrawal threshold in rats that were subjected to the sham operation, SNI without PRF (SNI group), or SNI plus either 45 V PRF or 60 V PRF (SNI + PRF-45 V and SNI + PRF-60 V groups, respectively). Mechanical allodynia was evaluated using dynamic plantar aesthesiometry. The data presented above were examined by ANOVA followed by Bonferroni test. D, day. #*P* < 0.05, ##*P* < 0.01 SNI, SNI + PRF-45 V, and SNI + PRF-60 V compared with the sham. *P < 0.05, **P < 0.01 SNI + PRF-45 V and SNI + PRF-60 V compared with SNI

**Fig. 3:**
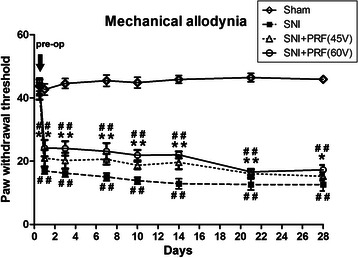
Paw withdrawal test of cold allodynia. Behavioral response in rats subjected to sham operation or SNI without PRF (SNI group) or SNI plus either 45 V PRF or 60 V PRF (SNI + PRF-45 V and SNI + PRF-60 V groups, respectively). Cold allodynia was evaluated by the acetone spray test. The data presented above were examined by ANOVA followed by Bonferroni test. D, day. #*P* < 0.05, ##*P* < 0.01 SNI, SNI + PRF-45 V, and SNI + PRF-60 V compared with the sham. *P < 0.05, **P < 0.01 SNI + PRF-45 V and SNI + PRF-60 V compared with SNI

No differences were observed between the four groups relative to the basal values of left hind paw behavioral tests in response to mechanical stimulus or acetone spray (*P* > 0.05; as listed in Figs 2 and 3). First, to investigate the “SNI effect”, we compared the three groups (SNI, SNI + PRF-45 V, and SNI + PRF-60 V) of rats with nerve injuries with the sham group and found that the sham group had a significantly lower paw withdrawal threshold (PWT) (*P*1 < 0.001; Fig. 2) and a longer painful-behavior duration (*P*1 < 0.001; Fig. 3) in two different behavioral measurements over 28 days. Second, we investigated the “treatment effect” by recording the left hind paw responses to mechanical stimulus and observed that both the SNI + PRF-45 V and SNI + PRF-60 V treatment groups had a significantly greater antiallodynic effect compared with the SNI group from days 1 to 28 after surgery (D1–D21, *P*2 < 0.001; D28, *P*2 < 0.05; Fig. 2). For the left hind paw responses in the acetone spray test, we found that rats in both the SNI + PRF-60 V and SNI + PRF-45 V groups had a significantly shorter painful-behavior duration than did SNI rats from days 1 to 28 (*P*2 < 0.001; Fig. 3). Third, during an exploration of the “dosage effect,” we observed no significant differences in the mechanical and cold hypersensitivity at all time points between the SNI + PRF-45 V and SNI + PRF-60 V groups (*P*3 > 0.05; Figs 2 and 3). No neuropathy was found in the sham-operated groups.

**Fig 4:**
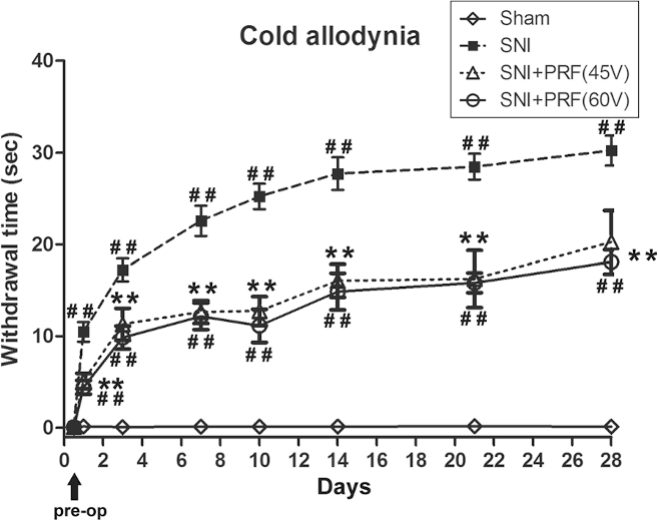
Effect of pulse repetition frequency on ERK1/2 expression in the spinal dorsal horn of rats with spared nerve injury. A. Representative Western blot showing upregulation of pERK1/2 and total ERK1/2 in the ipsilateral spinal cord at day 28 in various conditions (sham-operated control, SNI, and SNI + PRF-45 V). B. Histograms showing the results of a quantitative densitometric analysis of the pERK1/2 to ERK1/2 expression ratio. *n* = 4–6/group, *P < 0.05 compared with the sham control or PRF-45 V-treated rats. The data are expressed as the mean ± SEM. Differences between experimental groups were assessed by one-way ANOVA followed by Newman–Keuls multiple comparisons

The Western blot analysis of pERK1/2 and total ERK1/2 in the ipsilateral spinal cord at day 28 in the four groups (sham-operated control, SNI, SNI + PRF-45 V, and SNI + PRF-60 V) revealed an activation of ERK1 and ERK2 in the ipsilateral spinal dorsal horn of SNI rats, which was effectively reduced at day 28 in the SNI + PRF-45 V and SNI + PRF-60 V groups (Figs 4a, b). Figure 4a shows that total ERK1/2 expression exhibited no significant changes in all groups. In contrast, activated pERK1/2 was significantly increased after nerve injury in the SNI group, whereas a significant decrease in pERK1/2 levels was observed in the SNI + PRF-45 V and SNI + PRF-60 V groups at day 28. Figure 4b shows the ratio of the levels of pERK1/2 and total ERK1/2 (*n* = 4–6/group, *P < 0.05 compared with the sham control, PRF-45 V-treated, or PRF-60 V-treated rats). There was a 2.3-fold increase in the levels of activated p-ERK after SNI compared with the sham group, whereas the increase in ERK phosphorylation was inhibited by 44.74 % and 45.37 % after immediate PRF-45 V and PRF-60 V treatments compared with the SNI group, respectively.

## Discussion

In our study, immediate PRF treatment after SNI significantly alleviated the mechanical and cold allodynia in rats with SNI. After both the PRF-45 V and PRF-60 V treatments for 6 min, mechanical allodynia and cold hypersensitivity were reduced during the 28-day observation. Furthermore, we investigated the “dosage effect” of PRF treatment and found no significant differences in the mechanical and cold hypersensitivity at any time point between the SNI + PRF-45 V and SNI + PRF-60 V groups. PRF has been used widely for neuropathic pain management in clinical treatments, and a long-term effect has been observed after a single PRF application [34, 35]. In preclinical studies performed using a neuropathic pain model in the rabbit (induced by partial sciatic nerve ligation), both thermal and mechanical hyperalgesia were attenuated for 2 and 3 weeks after PRF application at 40–60 V for 8 min to the L5 and L6 dorsal roots [16]. In an L5 spinal nerve ligation neuropathic pain model in the rat, PRF attenuated mechanical allodynia for more than 32 days after a single PRF treatment at 25 V for 2 min adjacent to DRG [17]. Furthermore, PRF application at 45 V for 3 min on DRG yielded a reduction of mechanical hypersensitivity in rats with L5 spinal nerve ligation (SNL) and transaction [36]. In our study, immediate PRF was effective in alleviating the SNI-induced neuropathic pain. In particular, we observed that PRF treatment at 45 or 60 V for 6 min induced a similar antinociceptive effect for SNI that lasted up to 28 days.

In our examination, we found that allodynia developed at day 1 after SNI. Immediate PRF treatment after SNI resulted in a better anti-allodynia effect than that after 14 days of SNI (data not shown). It suggests that immediate PRF application takes only one times surgery and might inhibit severe immune response, providing better anti-allodynia effect with less undesirable side effects, such as adhesion and bleeding, than PRF treatment after 14 days of SNI. Furthermore, an animal study showed that even percutaneous application of PRF 50 V for 2 or 6 min provided a significant anti-allodynia effect in SNL rats [37]. Our study is the first to apply immediate treatment on the left sciatic nerve 0.3-0.4 cm proximal to the injured site in SNI rats. In addition, we further found that 60 V PRF treatment showed no better anti-allodynia effect than 45 V PRF during 28-day-observation. Accordingly, for PRF interventional treatments in radicular pain, it is advisable to choose the 45 V treatment, as the lower-voltage therapy produces less adverse effects while attaining similar benefits.

The application of PRF for the management of neuropathic pain should be considered carefully, as the mechanisms involved in the immediate versus the delayed phase may differ, and the immediate early phase of nerve injury is less understood. PRF for the management of clinical chronic neuropathic pain is common; however, sometimes it fails to provide a satisfactory effect, with the delayed intervention being one of the possible explanations for this finding. In the case of acute nerve injury, the acute inflammatory process without proper intervention often results in chronic neuropathic pain [38]. Lin *et al*. found that early PRF treatment suppressed the levels of proinflammatory cytokines via neuromodulation and immune modulation, such as the downregulation of spinal MAPK (ERK) activation [39]. Therefore, we assumed that an immediate or early PRF intervention may result in less neuroinflammation compared with a delayed PRF intervention.

In clinical practice, the application of immediate PRF therapy for the management of acute nerve injury might encounter several difficulties. Most patients will choose to undergo nerve reconstruction operation to restore neurological function. For those who cannot or will not undergo surgery, we propose an alternative, immediate/early PRF, to achieve a better effect, even though this study is still in the animal experiment stage. Nevertheless, to our knowledge, there is no clinical research-based investigation of the difference in antiallodynic effect between immediate and delayed PRF therapy after acute nerve injury. To translate our findings into future treatment strategies for developing neuropathic pain, we may choose to apply PRF treatment immediately, or combine one immediate and one delayed application of PRF to provide optimal results for patients.

The mechanisms underlying the effect of PRF treatment have not been well defined. It is thought to occur via a neuromodulatory effect that interferes with sensory neuron-specific molecules and gene expression involved in neuropathic pain development [36]. In other studies, RF stimulation at DRG altered synaptic transmission and cell morphology and induced Fos expression in the superficial dorsal horn, which suggests that this type of stimulation increases spinal neuron activity [18–20, 40]. The expression of ERK in DRG and the dorsal horn has been suggested to be a major target of mitogen-activated protein kinases (MAPKs) in the treatment of neuropathic pain. Suppression of ERK activation may be a promising therapeutic aim for the treatment of this type of pain [25–27]. Moreover, ERK was shown to be sequentially activated in different cell types in the dorsal horn of the spinal cord 21 days after the nerve injury in animals with SNL neuropathic pain [41]. Géranton *et al*. also demonstrated that SNI induces ERK activation in the dorsal horn of the spinal cord [42]. Similar to the results described here, activation of the phosphorylation of ERK1 and ERK2 in the ipsilateral spinal dorsal horn of SNI rats was found to be increased by 2.3-fold compared with the sham group. We also found that the increase in the phosphorylation of ERK1/2 observed after SNI was effectively inhibited (by 44.74 %) by immediate PRF therapy at 45 V at postsurgery day 28. Accordingly, we conclude that the analgesic effect of PRF may be attributed to its inhibitory effect on ERK activation in dorsal horn cells. Furthermore, in rats with neuropathic pain, the application of PRF on DRG attenuated SNL and transaction-induced neuropathic pain, with attenuation of microglial expression in the spinal dorsal horn [36]. Similarly, Lin *et al*. demonstrated that PRF inhibited ERK activation, which was detected on the third day after surgery [39]. Taken together, our results strongly suggest that immediate PRF treatment regulates ERK-mediated mechanisms in the spinal dorsal horn of SNI rats, which in turn reduces SNI-induced neuropathic pain. Accumulating evidence shows that the activation of MAPKs (p38, ERK, and JNK) can induce the synthesis of proinflammatory/pronociceptive mediators via distinct molecular and cellular mechanisms, resulting in the enhancement and prolongation of pain. Another study indicated that early application of PRF adjacent to the DRG significantly diminished nerve ligation-induced mechanical allodynia for 7 days and thermal hyperalgesia on postoperative days 3–7 by downregulating p38 and ERK activation [39]. However, the long-term effect of PRF on analgesia remains unknown. Interestingly, nerve injury activates ERK in microglia and astrocytes, as observed in the early (days) and late (weeks) phases, respectively [22]. Therefore, we investigated the long-term effects of PRF on ERK activation. Other investigators also found a similar long-term efficacy of continuous PRF-DRG in the treatment of chronic pain in humans, and reported that PRF-DRG treatment had an analgesic effect that ranged from weeks to over 6 months, affording over 50 % pain relief [43, 44]. Clearly, further investigation is required to understand the cell types in the dorsal horns that are involved in this effect and the role of ERK in the regulation of allodynia and hyperalgesia.

## Conclusions

Immediate application of PRF at 45 or 60 V on the proximal (0.3–0.4 cm) nerve injury site was an effective treatment for the management of neuropathic pain; the treatment was associated with an inhibition of ERK activity. For clinical translation, well-designed randomized controlled trials are required to identify the beneficial effect of PRF treatment proximal to the nerve injury site. Additional studies of the effect of PRF on the expression of ERK should be performed to provide evidences in treating neuropathic pain.

## Abbreviations

DPA: dynamic plantar aesthesiometery
DRG: dorsal root ganglion
ERK1/2: extracellular signal-regulated kinase 1/2
FOS: FBJ murine osteosarcoma viral oncogene homolog
MAPK: mitogen-activated protein kinases
PRF: pulsed radiofrequency
PWT: paw withdrawal threshold
SD: standard deviation
SEM: standard error of the mean
SNI: spared nerve injury
SNL: spinal nerve ligation
